# Supplementary material for: Maternal selenium deficiency was positively associated with the risk of selenium deficiency in children aged 6–59 months in rural Zimbabwe
Source: PLOS Glob Public Health. 2024 Jul 11;4(7):e0003376. doi: 10.1371/journal.pgph.0003376 (PMC11239066; doi:10.1371/journal.pgph.0003376)
Supplement: S1 Table — (DOCX) [file pgph.0003376.s001.docx]

**S1 Table**: Correlation between plasma Se concentration and acute phase proteins

| Variable | Test statistic | Plasma Se μg/L |
| --- | --- | --- |
| Children 6 - 59 months | | |
| CRP mg/L | **Pearson Correlation** | **0.009** |
|  | Sig. (2-tailed) | **0.817** |
|  | **n** | **693** |
| AGP g/L | **Pearson Correlation** | **-0.049** |
|  | Sig. (2-tailed) | **0.199** |
|  | **n** | **692** |
| Women of reproductive age (15 - 49 years) | | |
| CRP mg/L | **Pearson Correlation** | **-0.003** |
|  | Sig. (2-tailed) | **0.937** |
|  | **n** | **829** |
| AGP g/L | **Pearson Correlation** | **0.029** |
|  | Sig. (2-tailed) | **0.402** |
|  | **n** | **829** |

^CRP; C-reactive protein, AGP; alpha-1-acid glycoprotein. Adopted from (Mutonhodza^ *^et al.^*^, 2023). Linear regression^

Reference

- - - 1. Mutonhodza, B., Chagumaira, C., Dembedza, M. P., Joy, E. J., Manzeke-Kangara, M. G., Njovo, H., Nyadzayo, T. K., Lark, R. M., Kalimbira, A. A., Bailey, E. H., Broadley, M. R., And, T. M. M., & Chopera, P. (2023). A pilot survey of selenium status and its geospatial variation among children and women in three rural districts of Zimbabwe. July. <https://doi.org/10.3389/fnut.2023.1235113>
