# Supplementary material for: Maternal selenium deficiency was positively associated with the risk of selenium deficiency in children aged 6–59 months in rural Zimbabwe
Source: PLOS Glob Public Health. 2024 Jul 11;4(7):e0003376. doi: 10.1371/journal.pgph.0003376 (PMC11239066; doi:10.1371/journal.pgph.0003376)
Supplement: S2 Table — (DOCX) [file pgph.0003376.s002.docx]

**S2 Table: Correlation between child and maternal Se status stratified by child age group.**

| Child Se Status | Total  n (% of category) | Maternal Se status | | P-value^†^ |
| --- | --- | --- | --- | --- |
|  |  | ^‡^ Se adequate  n (%) | ^§^Se deficient  n (%) |  |
| 6-8 months |  |  |  |  |
| Deficient | 24 (96.0) | 7(100) | 17 (94.4) | >0.999 |
| Adequate | 1(4.0) | 0 | 1 (5.6) |  |
| 9-11 months |  |  |  |  |
| Deficient | 30 (100) | 7 (100) | 23 (100) |  |
| Adequate | 0 (0) | 0 (0) | 0 (0) |  |
| 12-17 months |  |  |  |  |
| Deficient | 85 (95.5) | 31 (93.6) | 54 (96.4) | 0.625 |
| Adequate | 4 (4.5) | 2 (6.1) | 2 (3.6) |  |
| 18-23 months |  |  |  |  |
| Deficient | 90 (94.7) | 21 (84.0) | 69 (98.6) | 0.016* |
| Adequate | 5 (5.3) | 4 (16.0) | 1 (1.4) |  |
| 24-35months |  |  |  |  |
| Deficient | 144 (96.6) | 45 (90.0) | 99 (100) | 0.004* |
| Adequate | 5 (3.4) | 5 (10.0) | 0 (0) |  |
| 36-47 months |  |  |  |  |
| Deficient | 119 (95.2) | 29 (85.3) | 90 (98.9) | 0.006* |
| Adequate | 6 (4.8) | 5 (14.70 | 1 (1.1) |  |
| 48-59 months |  |  |  |  |
| Deficient | 111 (96.5) | 31 (91.2) | 80 (98.8) | 0.077 |
| Adequate | 4 (3.5) | 3 (8.8) | 1 (1.2) |  |

^§ Plasma Se concentration level < 70 μg/L; ‡Plasma Se concentration ≥70 μg/L; †P value *significant at p < 0.05 from Pearson’s Chi square test.^

Reference

- - - 1. Mutonhodza, B., Chagumaira, C., Dembedza, M. P., Joy, E. J., Manzeke-Kangara, M. G., Njovo, H., Nyadzayo, T. K., Lark, R. M., Kalimbira, A. A., Bailey, E. H., Broadley, M. R., And, T. M. M., & Chopera, P. (2023). A pilot survey of selenium status and its geospatial variation among children and women in three rural districts of Zimbabwe. July. <https://doi.org/10.3389/fnut.2023.1235113>
