# Supplementary material for: Maternal selenium deficiency was positively associated with the risk of selenium deficiency in children aged 6–59 months in rural Zimbabwe
Source: PLOS Glob Public Health. 2024 Jul 11;4(7):e0003376. doi: 10.1371/journal.pgph.0003376 (PMC11239066; doi:10.1371/journal.pgph.0003376)
Supplement: Attachment — Submitted filename: Response to the reviewers.docx [file pgph.0003376.s004.docx]

REBUTTAL

PGPH-D-24-00427

Maternal selenium deficiency was positively associated with the risk of selenium deficiency in children aged 6-59 months in rural Zimbabwe

PLOS Global Public Health

5. Review Comments to the Author

Please use the space provided to explain your answers to the questions above. You may also include additional comments for the author, including concerns about dual publication, research ethics, or publication ethics. (Please upload your review as an attachment if it exceeds 20,000 characters)

Rebuttal: The authors thank the reviewer for the valuable comments and suggestions. Revisions have been addressed as highlighted in the responses below.

Reviewer #1: Thank you for the opportunity to review the paper titled: “Maternal selenium deficiency was positively associated with the risk of selenium deficiency in children aged 6-59 months in rural Zimbabwe”.

The paper is well written and shares some interesting information about selenium concentrations in human specimens and its correlates.

Recommend major revisions.

Line 49: Can you explain why? How and why does HIV exacerbate Se deficiency in SSA?

Response:

“Selenium deficiency is associated with the pathogenicity of several viruses, including human immunodeficiency virus (HIV) (Guillin OM, Vindry C, Ohlmann T, Chavatte L. Selenium, selenoproteins and viral infection. Vol. 11, Nutrients. 2019. 1–33 p.).

The African region is the most affected, with 25.7 million people living with HIV in 2018 (World Health Organization (WHO). HIV/AIDS. WHO. Geneva, Switzerland. 2018).

Viral infection simultaneously increases the demand for micronutrients including Se and causes their loss, exacerbating deficiency (Guillin OM, Vindry C, Ohlmann T, Chavatte L. Selenium, selenoproteins and viral infection. Vol. 11, Nutrients. 2019. 1–33 p).

An explanation has been added to the text Line 49-50

“Viral infection simultaneously increases the demand for micronutrients and causes their loss, exacerbating deficiency”.

Lines 134-135: For inflammation adjustment and Se, can you kindly explain why you have specifically noted negative correlations Is this expected based on the literature, if so, please cite biological plausibility and theory to explain why? Would you not apply correction if the relationships were positive? Kindly define the negative acute phase response in a little more detail with relevant citations.

Response:

The literature points to a negative correlation;

The acute phase response or infection can lower the levels of Se, GPX3 activity, and SELENOP ( Couser, N., & Gucsavas-Calikoglu, M. (2017). Chapter 8. Mitochondrial disorders. In Biomarkers in Inborn Errors of Metabolism. Elsevier Inc. <http://dx.doi.org/10.1016/B978-0-12-802896-4.00008-0>).

Galloway et al. (2000) noted a decline in Se concentration by up to 10 % during an acute response (24hrs)( Galloway, P., McMillan, D. C., & Sattar, N. (2000). Effect of the inflammatory response on trace element and vitamin status. Annals of Clinical Biochemistry, 37(3), 289–297. <https://doi.org/10.1258/0004563001899429>),

and a 40-60 % decline was reported by Thurnham et al. (2015) in chronic or long-term (3-10 days) inflammation ( Thurnham, D. I. (2015). Inflammation and Biomarkers of Nutrition. Sight and Life Magazine: Frontiers in Nutrition, 2015(1), 51–59. <https://doi.org/10.52439/bszl2497>)

However, the concern has been noted and the statement has been adjusted to read Lines 145-147

“No significant correlation was observed between plasma Se concentration and any of the inflammation biomarkers measured (**Supplementary material 1)**, thus no correction was applied for inflammation (50,54)”

A supplementary file showing the results has been attached. Please note that the same file was provided in the reference (35) indicated in line 147. It had been excluded here for duplication reasons.

**Supplementary material 1**: Correlation between plasma Se and acute phase proteins

| Variable | Test statistic | Se μg / L |
| --- | --- | --- |
| Children 6 - 59 months | | |
| CRP mg / L | **Pearson Correlation** | **0.009** |
|  | Sig. (2-tailed) | **0.817** |
|  | **n** | **693** |
| AGP g / L | **Pearson Correlation** | **-0.049** |
|  | Sig. (2-tailed) | **0.199** |
|  | **n** | **692** |
| Women of reproductive age (15 - 49 years) | | |
| CRP mg /L | **Pearson Correlation** | **-0.003** |
|  | Sig. (2-tailed) | **0.937** |
|  | **n** | **829** |
| AGP g / L | **Pearson Correlation** | **0.029** |
|  | Sig. (2-tailed) | **0.402** |
|  | **n** | **829** |

Lines 1222-135: Can you please define what % of your blood samples were below the LOD/LOQ? How did you handle thresholded data? Was any imputation used to account for values < LOD (i.e., low (0), medium (1/2 LOD), high (LOD) imputation? If no imputation was used, kindly define what % of your samples were below the LOD/LOQ at the least and how you handled these observations?

Response

All sample observations were above the LOD/LOQ limits.

This has been highlighted in the text Lines 141-142

“All the sample observations were above the LOD/LOQ limits”.

Line 146: Why did you use a p < 0.10 cut-off to define significance?

Response

The level of significance was at <0.05, the 0.10 is the removal criteria set as the fixed decision rule to determine variable significance. The wording has been changed to match the explanation below;

The removal criterion (pR) is the p-value (i.e. probability value) used to eliminate insignificant independent variables. If a variables p-value is equal to or greater than this number it will be eliminated from the model. The entry criterion (p_E_) value determines which independent variables will be included in the model. If a variable’s p-value is less than this value then it will be entered into the model..

The wording has been changed to read Lines 157-159

“further analyzed using logistic regression, with the removal criterion (pR) value set at 0.10 and 0.05 as the entry criterion (p_E_) value by the enter method to establish predictors of Se deficiency. All statistical measures were at 95 % confidence interval”.

Can you kindly also look at your data using linear regression i.e., continuous plasma Se to define how your covariates respond when using the variable without dichotomizing it.

Response

The covariates are not continuous and the objective of the study to establish the determinants of Selenium deficiency warrants dichotomization of the continuous plasma data into two groups (sufficient vs. deficient) from the start. Using linear regression i.e., continuous plasma Se to define how your covariates respond when using the variable without dichotomizing it may not be of much benefit to the current study.

Can you also show your correlation analyses in some form of a correlation matrix so the reader can see what your coefficients looked like, before proceeding to logistic regression?

Response

The data presented in Tables 1 and 2 are a collation of individual correlation matrices for the different variables. Only those found to be significant from these tables were then analyzed by logistic regression to confirm the predictive influence.

For example, in Table 1,

Table 1: These data seem to be clearly pointing to soil insufficiency for Se. Given Se is primarily found in soil and insufficiency in concentrations in the soil will lead to population deficiency, it is very clear that there should be more work done to examine crop concentrations of Se to define whether there is generalized insufficiency/deficiency in these regions. This may be particularly relevant given homestead farming may not regulate use of fertilizers and/or biofortified crops. You have commented on this in your discussion, but you may want to discuss limitations in more detail later on in your text and/or suggest how you would extend these analyses in terms of next steps which could involve examining soil, crop and dietary Se concentrations (whole foods using total diets data for e.g. since deficiency is so high in the population).

Response

More detail as alluded has been incorporated into the discussion

Lines 322-326 now read

“Cognisant of that, soil Se concentrations are the primary driver of population Se status, future research should involve the examination of soil, crop, and dietary Se concentrations (for example whole foods using total diet data) to define whether there is a generalized deficiency in these regions. This may be particularly relevant given homestead farming may not regulate the use of fertilizers and/or biofortified crops”.

Table 1/Lines 185-186: There may be a need to discuss in the introduction, what foods in the typical Zimbabwean diet are rich in Se and why you are seeing such strong significant associations between production of these staples and Se deficiency – are these food typically low in Se (globally and in Zimbabwe and if so why?).

Response

Background information has been added to the introduction Lines 54-62

“Diets in sub-Saharan Africa consist primarily of carbohydrates (16). In many parts of Africa, rural diets are frequently monotonous, consisting mainly of starchy foods such as grains, tubers, and roots but with limited or negligible intake of animal-source foods (17). The consumption of animal foods such as meat, poultry, and fish is limited, mainly because of economic, cultural, and religious constraints (15). Plants can be classified into three main groups based on the Se concentrations in their tissues; non-accumulators, accumulators, and hyperaccumulators (18). Non-accumulating plants such as grains and grasses contain lower concentrations of Se (18,19). Maize grain in sub-Saharan African countries has a sub-optimal Se concentration of ˂ 50 µg/kg dry mass (2) and is not likely to meet human requirements (20).”

Lines 191-192: Can you look at dietary data to examine how they correlate to Se deficiency and Se concentrations using linear and logistic regression.

Response

The data presented is for crops commonly grown for consumption, not dietary data. Dietary data collection has been suggested for future steps. The words “production of” have been added to the text to avoid confusion.

It now reads lines 202-203

“production of maize (p <0.001), production of cowpeas (p <0.001), production of groundnuts (p <0.001), production of sweet potatoes (p <0.001), production of sugar beans (p <0.001) and production of onions”

Lines 200-202: This sentence needs to be clarified. Can you please include the accompanying Se deficiency % in mother-child dyads?

Response

This has been addressed Line 213-215

“Child Se status was significantly associated with maternal Se status (p <0.001). The proportion of Se-deficient children was higher (98.7 %) in Se-deficient mothers compared to Se-adequate mothers (90.2 %) Table 3”.

Table 4: For the overweight/obese cells before line 213, wouldn’t the 0.087 p-value be significant as per the criteria you have described in your methods.

Response

This has been addressed

The level of significance is p < 0.05, this has been clarified in methods as indicated above.

Table 5: Please define your reference values for table 5

Response

This has been addressed in Lines 240-241

**“Notes**: Selenium deficient mother; Plasma Se concentration <70 μg / L; Selenium adequate mother; Plasma Se concentration ≥70 μg / L, †P value from multiple logistic regression analysis, *Significant at P<0·05., at 0.10 probability of stepwise removal using the Enter regression model.”

Lines 228-238: The issue of sex specific differences needs to be explained further.

Selenium Metabolism, Regulation, and Sex Differences in Mammals | SpringerLink

Selenium is concentrated in male gonads which could possibly explain why male children had lower deficiency had than female children – given what is known of selenium saturation in the human body. Kindly refer to the text above for more. Could you attribute your findings to sex differences in infant and young child feeding practices/breastfeeding behaviours and gendered preferences for male children, if culturally relevant? Can you comment on diet quality and differences between male vs. female children if relevant?

Response

These have been considered Lines 265-275

“sexual dimorphic regulation of Se metabolism and selenoprotein expression, namely the trans-selenation pathway by sex hormones strongly implies that selenomethionine metabolism and its consequent selenocysteine formation and availability for selenoprotein synthesis are not the same in both sexes (61). Selenium is concentrated in male gonads which could explain why male children had lower deficiency than female children (62); or gender-based vulnerabilities (63,64) influencing food intake (64). In India, girls were more likely to be neglected than boys to receive nutritious diets (65), female children were breastfed for a shorter duration and had lower consumption of dairy food compared to male children (66). These differences can be attributed to gender incongruence in the intra-household food allocation for children, which is affected by cultural norms in society and women’s empowerment in households (67).”.

Line 252-253: Sentence is incomplete.

Response

The sentence has been rephrased, it now reads Line 278-279

“Sex variabilities could be implemented in MNP supplementation, as seen in growth monitoring, where growth charts target individual sexes (44)”.

Line 257-261: You may consider looking into how placental transfer of Se in utero can affect selenium sufficiency/insufficiency postpartum. Since you see clear statistically significant trends in maternal and child Se deficiency, you may want to examine diet quality which is a likely confounder, given it would affect Se status in mothers and children. Since you have younger children in this study (6-12 months), you may consider stratifying children by age group to see if the younger children have stronger correlation with maternal Se status compared to the older children. Additionally, you may consider discussing birth order of the children involved.

Response

The study was for the characterization of determinants to guide further research, the suggested variables that were not considered in the current study have been recommended for future research in the discussion section Lines 307-309

“Further research into placental transfer of Se in utero, diet quality, and birth order which can affect Se sufficiency/insufficiency postpartum is recommended to validate the findings of the current study.”

The data was stratified by child age group as suggested and presented as supplementary material 2.

The results were presented in Lines 215-218

“with older children being significantly more Se-deficient compared to the younger children. Among the Se-deficient mothers, children aged 6-8 months were the least Se-deficient (94.4 %, p >0.999) while those in the age range 24-35 months were the most Se-deficient (100 %, p = 0.004) **Supplementary material 2”**.

And discussion lines 290-292

“Our results indicated a lower prevalence of Se deficiency in younger children and a higher prevalence in older children among Se-deficient mothers this can be attributed to the protective effect of breastfeeding”.

**Supplementary material 2: Child and maternal Se status stratified by child age group.**

| Child Se Status | Total  n (% of category) | Maternal Se status | | P-value^†^ |
| --- | --- | --- | --- | --- |
|  |  | ^‡^ Se adequate  n (%) | ^§^Se deficient  n (%) |  |
| 6-8 months |  |  |  |  |
| Deficient | 24 (96.0) | 7(100) | 17 (94.4) | >0.999 |
| Adequate | 1(4.0) | 0 | 1 (5.6) |  |
| 9-11 months |  |  |  |  |
| Deficient | 30 (100) | 7 (100) | 23 (100) |  |
| Adequate | 0 (0) | 0 (0) | 0 (0) |  |
| 12-17 months |  |  |  |  |
| Deficient | 85 (95.5) | 31 (93.6) | 54 (96.4) | 0.625 |
| Adequate | 4 (4.5) | 2 (6.1) | 2 (3.6) |  |
| 18-23 months |  |  |  |  |
| Deficient | 90 (94.7) | 21 (84.0) | 69 (98.6) | 0.016* |
| Adequate | 5 (5.3) | 4 (16.0) | 1 (1.4) |  |
| 24-35months |  |  |  |  |
| Deficient | 144 (96.6) | 45 (90.0) | 99 (100) | 0.004* |
| Adequate | 5 (3.4) | 5 (10.0) | 0 (0) |  |
| 36-47 months |  |  |  |  |
| Deficient | 119 (95.2) | 29 (85.3) | 90 (98.9) | 0.006* |
| Adequate | 6 (4.8) | 5 (14.70 | 1 (1.1) |  |
| 48-59 months |  |  |  |  |
| Deficient | 111 (96.5) | 31 (91.2) | 80 (98.8) | 0.077 |
| Adequate | 4 (3.5) | 3 (8.8) | 1 (1.2) |  |

^§ Plasma Se concentration level < 70 μg/L; ‡Plasma Se concentration ≥70 μg/L; *Significance level P <0·05; †P value significant at p<0.05 from Pearson’s Chi square test.^

Line 257-261: What is known of Se concentrations in the 6-12 month age group, given you are not discussing a supplementation study here? We know Se concentrations decrease during pregnancy and at delivery and then level off, can you comment on this matter and how it could be impacting the strong correlations you see in mother-infant dyads if relevant?

Response

This has been addressed Lines 299-303

“Selenium concentrations were reported to be low in women of reproductive age in the United Kingdom, decreasing further during pregnancy, thus resulting in low plasma and placental antioxidant enzyme activities (80). Multiple micronutrient supplementation during pregnancy indicated an upward trend in Se concentration across semesters (81).”

Line 285-287: You may consider examining whether mycotoxin contamination in crops in Zimbabwe is responsible for low Se concentrations. T-2 toxin co-occurs with Se deficiency in soil and could be an explanatory factor if relevant in your context, in addition to other mycotoxins present in crops including maize and groundnuts. There may be other mycotoxins of relevance in addition to T-2 toxin. Kindly briefly examine the literature to see if this is a relevant explanatory factor. You may consider examining the work of Laura Smith and Francis Ngure (Zwitambo) for more.

Response

The literature examined emphasized the detrimental effects of combined T2 -toxin exposure and Se deficiency this has been incorporated into the discussion

Lines 327-331

Further to this, maize grain in Zimbabwe has been implicated with mycotoxin contamination mainly *fusarium* (91). *Fusarium* produces T-2 toxin as a secondary metabolite whose synergistic effects with Se deficiency pose potential detrimental health hazards to the Se-deficient population (92). Investigation of this correlation might be warranted.

6. PLOS authors have the option to publish the peer-reviewed history of their article (what does this mean?). If published, this will include your full peer review and any attached files.

Do you want your identity to be public for this peer review? If you choose “no”, your identity will remain anonymous but your review may still be made public.

For information about this choice, including consent withdrawal, please see our Privacy Policy.

Reviewer #1: No

[NOTE: If reviewer comments were submitted as an attachment file, they will be attached to this email and accessible via the submission site. Please log into your account, locate the manuscript record, and check for the action link "View Attachments". If this link does not appear, there are no attachment files.]

While revising your submission, please upload your figure files to the Preflight Analysis and Conversion Engine (PACE) digital diagnostic tool, https://pacev2.apexcovantage.com/. PACE helps ensure that figures meet PLOS requirements. To use PACE, you must first register as a user. Registration is free. Then, login and navigate to the UPLOAD tab, where you will find detailed instructions on how to use the tool. If you encounter any issues or have any questions when using PACE, please email PLOS at figures@plos.org. Please note that Supporting Information files do not need this step.
